# MDI or nebulization in moderate to severe COVID-19 disease with COPD: which one is better?

**DOI:** 10.1186/s42077-021-00148-4

**Published:** 2021-03-24

**Authors:** Geetanjali T. Chilkoti, Prakash G. Gondode, Spriha S. Tiwari

**Affiliations:** 1 Department of Anesthesiology and Critical Care, University College of Medical Sciences and GTB Hospital, New Delhi, India; 2 Department of Anesthesiology and Critical Care, All India Institute of Medical Sciences, Nagpur, Maharashtra India

To the Editor,

Aerosol bronchodilator therapy via nebulization using a nebulizer or metered dose inhaler (MDI) with a spacer device or dry powder inhaler is the mainstay in the treatment of COPD. We, herein, discuss two such cases with corresponding concerns related to various modes of bronchodilation in moderate to severe COVID-19 patients with COPD.

Two COVID-19 patients (60 years/M and 55 years/M) with COPD presented with worsening fever, cough, and shortness of breath for around 10 days. Chest radiographs revealed mid and lower lung zone involvement in both. Both received tazobactam-piperacillin for a period of 10 days and 12 days respectively, along with teicoplanin, ivermectin, hydroxychloroquine, enoxaparin, dexamethasone, salmeterol MDI, and multivitamin, and received oxygen via high FiO_2_ non-rebreathing mask and maintained 92–94% oxygen saturation and 24–32 breaths/min (FiO_2_ delivered was 0.8–1). We witnessed their inability to achieve an optimal peak inspiration with MDI and thus increased the dose to @ 4–6 puffs. Following no improvement, nebulization was started. Jet nebulizer was used, and levosalbutamol was used. The device contained mouthpiece with expiratory valve, but there was no filter available on the outlet end. Nevertheless, extreme caution was taken in ensuring safety of the health care workers, as all donned PPE. With improvement in peak inspiratory flow rate (more than 60 L/min), the MDIs were reinstituted. The pulmonary symptoms improved and both the patients were discharged subsequently. The total duration of stay in hospital was 17 days and 22 days respectively. Both patients got intensive nebulization therapy QID for a period of 4–5 days. A written informed consent for publication was obtained from both the patients.

## Discussion

Bronchodilator delivery by MDI or wet nebulizer has been found to be equivalent in the acute treatment of adults with airflow obstruction (Turner et al., 1997). A Cochrane review (van Geffen et al., 2016) observed no significant difference in FEV_1_ at 1 h after dosing between nebulization and MDI; however, an improved FEV_1_ trend was observed with nebulization (van Geffen et al., 2016).

In critically ill patients, nebulization is preferred over MDI or DPI (dry powder inhaler) as the latter needs an optimal peak inspiratory flow rate (approx. 60 L/min) which is often compromised in critical illness. During COVID pandemic, bronchodilation via nebulization has got serious concern, i.e., the nebulizers produce small- and medium-size aerosol range and can disperse viral particles in exhaled air > 0.8 m from the patient and remain airborne for more than 30 min (Hanak & Chatwin, 2010), thus a potential exposure threat to the health care workers (HCW). It must rather be provided in isolation rooms with negative pressures and HCW providing care should don quality PPE (grade 3 personal protective equipment).

The expert consensus guidelines recommend replacing nebulization with MDIs in COVID-19 disease; however, there are no dogmatic guidelines or protocols in this context (Abrams & Szefler, 2020). As far as pathophysiology is concerned, COPD is an obstructive airway pathology characterized by expiratory airflow limitation due to chronic inflammation of the large central airways, peripheral bronchioles, and destruction of lung parenchyma. On the contrary, in COVID-19, Mu X et al. observed it to be primarily a restrictive ventilatory defect along with impairment of diffusion capacity as reflected by the pulmonary function tests in 110 discharged survivors with COVID 19 (Mo et al., 2020). Therefore, COVID patients with COPD may present with mixed pattern (obstructive and restrictive) which may affect the performance of the aforementioned modes of bronchodilation.

We emphasize the need for assessing the risk-benefit ratio related to the safety of HCW with the use of MDI plus spacer versus the risk of clinical deterioration by avoiding nebulization in patients with COVID-19 disease with COPD. We also recommend the need for further research and evidence-based concrete guidelines in context to the favorable mode of inhaled bronchodilator in COVID-19 disease.

## Data Availability

Not applicable.

## Abbreviations

MDI: Metered dose inhaler
COVID: Coronavirus disease
COPD: Chronic obstructive pulmonary disease
FiO2: Fraction of inspired oxygen
FEV1: Forced expiratory volume in 1 s
DPI: Dry powder inhaler
HCW: Health care worker
